# The Dysregulation of SOX Family Correlates with DNA Methylation and Immune Microenvironment Characteristics to Predict Prognosis in Hepatocellular Carcinoma

**DOI:** 10.1155/2022/2676114

**Published:** 2022-04-13

**Authors:** Sha Qin, Gaoming Liu, Haoer Jin, Xue Chen, Jiang He, Juxiong Xiao, Yan Qin, Yitao Mao, Luqing Zhao

**Affiliations:** ^1^Department of Pathology, Xiangya Hospital, Central South University, Changsha, Hunan, China; and Department of Pathology, School of Basic Medical Science, Xiangya School of Medicine, Central South University, Changsha, Hunan, China; ^2^Xiangya School of Medicine, Central South University, Changsha, Hunan, China; ^3^Early Clinical Trial Center, Hunan Cancer Hospital and The Affiliated Cancer Hospital of Xiangya School of Medicine, Central South University, Changsha, Hunan, China; ^4^Center for Molecular Medicine, Xiangya Hospital, Central South University, Changsha, Hunan, China; ^5^Department of Radiology, Xiangya Hospital, Central South University, Changsha, Hunan, China; ^6^National Clinical Research Center for Geriatric Disorders, Xiangya Hospital, Central South University, Changsha, Hunan, China

## Abstract

**Background:**

Due to the molecular heterogeneity of hepatocellular carcinoma (HCC), majority of patients respond poorly among various of therapy. This study is aimed at conducting a comprehensive analysis about roles of SOX family in HCC for obtaining more therapeutic targets and biomarkers which may bring new ideas for the treatment of HCC.

**Methods:**

UALCAN, Kaplan Meier plotter, cBioPortal, STRING, WebGestalt, Metascape, TIMER 2.0, DiseaseMeth, MethSurv, HPA, CCLE database, and Cytoscape software were used to comprehensively analyze the bioinformatic data.

**Results:**

SOX2, SOX4, SOX8, SOX10, SOX11, SOX12, SOX17, and SOX18 were significantly differentially expressed in HCC and normal tissues and were valuable for the grade and survival of HCC patients. In addition, the gene alterations of SOX family happened frequently, and SOX4 and SOX17 had the highest mutation rate. The function of SOX family on HCC may be closely correlated with the regulation of angiogenesis-related signaling pathways. Moreover, SOX4, SOX8, SOX11, SOX12, SOX17, and SOX18 were correlation with 8 types of immune cells (including CD8+ T cell, CD4+ T cell, B cell, Tregs, neutrophil, macrophage, myeloid DC, and NK cell), and we found that most types of immune cells had a positive correlation with SOX family. Notably, CD4+ T cell and macrophage were positively related with all these SOX family. NK cells were negatively related with most SOX family genes. DNA methylation levels in promoter area of SOX2, SOX4, and SOX10 were lower in HCC than normal tissues, while SOX8, SOX11, SOX17, and SOX18 had higher DNA methylation levels than normal tissues. Moreover, higher DNA methylation level of SOX12 and SOX18 demonstrated worse survival rates in patients with HCC.

**Conclusion:**

SOX family genes could predict the prognosis of HCC. In addition, the regulation of angiogenesis-related signaling pathways may participate in the development of HCC. DNA methylation level and immune microenvironment characteristics (especially CD4+ T cell and macrophage immune cell infiltration) could be a novel insight for predicting prognosis in HCC.

## 1. Introduction

Hepatocellular carcinoma (HCC), which accounts for 75% to 85% of primary liver cancer, is one of the leading causes of cancer-related deaths worldwide [1]. The current therapeutic strategies for HCC mainly include drug therapy and nondrug therapy. Among the main nonpharmacological treatments are HCC resection, liver transplantation, transarterial chemoembolization (TACE), percutaneous ablation, and chimeric antigen receptor engineered T-cell immunotherapy (CAR-T), while some small molecule targeted drug therapies, such as sorafenib, lenvatinib, regorafenib, and the monoclonal antibodies such as nivolumab are used as the mainstay of drug therapy for HCC. However, the survival rate of HCC patients is still unsatisfied, although more and more efforts for HCC therapy have been made in recent years [2]. The identification of novel biomarkers are urgent for the molecular-targeted therapy agents. With the development of epigenetics, multiple studies have now focused on the exploration of potential prognostic epigenetic markers, which include DNA methylation, histone acetylation, chromatin remodeling, and noncoding RNAs. For instance, histone acetylation made a great contribution to predict disease. One recent research demonstrated that in PBMC, low H3K27 acetylation of SF1 could act as a biomarker for the adrenal insufficiency of steroid synthesis [3]. Moreover, histone code reader protein was reported to be associated with abnormal chromatin regulation in cancer. Heterochromatin protein 1 could serve as a potential biomarker for cancer prognosis by recognizing histone H3 lysine 9 methylation as well as affect chromatin biology [4]. SMARCC1, a SWI/SNF chromatin remodeling factor, was highly expressed in HCC tissues, which predicted poorly prognoses and may become a novel biomarker to predict survival in HCC patients [5]. Additionally, ARID1A, a key component of the SWI/SNF chromatin remodeling complexes, was related to the resistance to EGFR-TKIs in non-small cell lung cancer, and it could serve as a novel biomarker [6]. Noncoding RNAs, which had tissue-specific patterns of expression, were reported to be potential cancer biomarkers, and they played a role in regulating chromatin stability, mRNAs translation, and the functional regulation of membraneless nuclear bodies [7]. For example, lncRNA THEMIS2-211, an exosomal biomarker, promoted the growth and metastasis of HCC by functioning as a competing endogenous RNA [8]. In this study, we mainly focused on DNA methylation.

DNA methylation alteration was tissue-specific and could regulate gene transcription in cell proliferation and survival. The gene expressions as well as DNA methylation analyses contribute to identify cancer markers. MCM2 and NUP37 are promising prognostic biomarkers, and the demethylation of enhancer could regulate the expression of these two genes in HCC. That is to say, MCM2 and NUP37 may be potential targets for epigenetic therapy in HCC patients [9]. Through matching gene expression profiles and the promoter methylation data in TCGA database, Chen et al. found that TIPIN was the gene with discrepant expression as well as the gene with differential promoter methylation in HCC, and it could be a potential novel epigenetic prognostic biomarker [10]. In addition, in breast cancer, PCDHB15, a potential tumor suppressor, was reported to be epigenetically silenced via DNA promoter methylation, and it might be an epigenetic biomarker for the diagnosis and prognosis of breast cancer [11]. Lietz and his colleges discovered that the recurrence and survival of osteosarcoma was associated with genomic methylation, and the relative genomic hypomethylation could be strongly predictive of the response to chemotherapy [12]. In another research, the hypermethylation of BRCA2 promoter could act as a biomarker for the leukemic transformation of myeloproliferative neoplasms [13]. However, accumulated researches reported that most HCC patients lack of efficient biomarkers for early detection or screening [14]. Therefore, developing more effective molecular biomarkers (for example, focusing on DNA methylation) will contribute to the early diagnosis and treatment of HCC patients.

Sex-determining region Y (Sry)-box-containing (SOX) family members (including SOX1, SOX2, SOX3, SOX4, SOX5, SOX6, SOX7, SOX8, SOX9, SOX10, SOX11, SOX12, SOX13, SOX15, SOX17, and SOX18) are transcription factors with a significant role such as tumor growth and invasion in various of cancers [15–19]. Moreover, recent studies indicated that SOX family had a potential to become novel biomarkers for cancer diagnosis and prognosis [20–22]. However, among all SOX family genes, only SOX13 and SOX18 seem to be investigated more in HCC at present. For example, Feng et al. revealed the clinical significance and biological function of SOX13 in HCC. And the results showed that the upregulation of SOX13 could maintain cancer stem-like properties in HCC cells and was associated with the poor differentiation, metastasis, and recurrence of HCC patients [23, 24]. In addition, the high expression of SOX18 promoted HCC metastasis by upregulating metastasis-related genes and was reported positively correlated with poor tumor differentiation and poor prognosis [25].

In this paper, we conducted a comprehensive bioinformatics analysis of the SOX family in HCC based on TCGA database. In addition, we explored the expression patterns, prognostic values, mutation situation, functional enrichment analysis, immune cells infiltration, and methylation levels of the SOX gene in HCC, which may offer some new opportunities for targeted therapies in HCC.

## 2. Materials and Methods

### 2.1. Data Collection

RNA-seq data and clinical information were obtained from TCGA database, and we used all the available RNA-seq data and clinical information (including age, gender, tumor grade, individual cancer stage, and nodal metastasis status) about normal and HCC samples from TCGA database, which includes transcriptome data from 371 HCC samples and 50 noncancerous samples [26] (Table 1). The website was https://portal.gdc.cancer.gov/.

### 2.2. Gene Expression Analysis

UALCAN database is an online website to analyze relative expression of queried genes across tumor and normal samples [27]. The website is http://ualcan.path.uab.edu/index.html. We analyzed the expression of SOX family in HCC patients with different clinical features in UALCAN based on type, stage, and nodal metastasis. The differences in transcriptional expression were compared by Student's *t*-test, and *P* < 0.05 were considered as statistically significant.

Human Protein Atlas (HPA) database can be used to analyze the relationship between protein-coding genes for cancer and clinical outcomes, thus exploring the impact of individual proteins on clinical outcomes at genome-wide area [28]. The website is https://www.proteinatlas.org. We used this database to obtain the protein expression level of key genes in HCC and normal liver tissue.

Cancer Cell Line Encyclopedia (CCLE) database can be used to study genetic variants, candidate targets, small molecules, and biotherapeutics and to identify novel marker-driven cancer dependencies, which can also reveal potential targets for cancer drugs and related biomarkers [29]. In this study, cell line mRNA expression matrix of HCC was obtained from the CCLE dataset. The website is https://portals.broadinstitute.org/ccle.

### 2.3. Survival Analysis

To evaluate the prognostic value of the dysregulated SOX genes, we estimated their OS and PFS in HCC by using Kaplan-Meier analysis methods [30]. We considered *P* < 0.05 as statistically significant. Kaplan-Meier plotter was available from http://kmplot.com/analysis/. Then, combined with the RNA-seq data and clinical features, we used these combined genes for further analysis.

### 2.4. Genetic Mutation Analysis

cBioPortal database reduces molecular profiling data into understandable genetic, epigenetic, gene expression, and proteomic events and could provide the summaries of gene-level data from multiple platforms [31]. cBioPortal was available from https://www.cbioportal.org. In this study, cBioPortal was used to analyze the genome map of the SOX family in HCC and to obtain the mutation and mRNA expression data. Besides, the co-expression genes of SOX family from cBioPortal were downloaded for further analysis.

### 2.5. Protein-Protein Interaction Analysis

We used STRING database to get protein-protein interaction and computational predictions information [32]. STRING was available from https://string-db.org/ . In this study, STRING database was used to analyze possible protein-protein interactions.

Cytoscape was especially suit for humans and model organisms, when used in conjunction with large databases of protein-protein, protein-DNA, and genetic interactions [33]. Then, we used Cytoscape to integrate co-expression genes of SOX family that obtained from cBioPortal. The co-expression genes were integrated from top 20 genes that had a high spearman's correlation value (> 0.4) with SOX family. Besides, we used it to figure out top 10 hub genes of these co-expression genes.

### 2.6. Functional Enrichment Analysis

We used WebGestalt and Metascape to conduct functional enrichment analysis of SOX family. WebGestalt supports three kinds of enrichment analysis, including over-representation analysis (ORA), gene set enrichment analysis (GSEA), and network topology-based analysis (NTA) [34]. And WebGestalt was available from http://www.webgestalt.org/. Metascape is a popular portal, which combines functional enrichment, interactome analysis, gene annotation, and membership [35]. Metascape was available from https://metascape.org/gp.

### 2.7. Evaluation of Tumor Infiltrating Immune Cells

TIMER2.0 used computational algorithms to infer immune cell composition from bulk tumor transcriptome profiles, thus providing insight into tumor-immune interactions [36]. TIMER2.0 was available from http://timer.cistrome.org/. In this study, we used TIMER2.0 to depict the relationship between SOX family and immune cells including CD8+ T cell, CD4+ T cell, T cell regulatory (Tregs), B cell, neutrophil, macrophage, myeloid dendritic cell (DC), and nature killer (NK) cell. All of these data had a purity adjustment process.

### 2.8. Methylation Analysis

DiseaseMeth is a database that is available for analysis DNA methylation data in human cancers, and now it enables online automated identification of DNA methylation abnormalities in human disease [37]. DiseaseMeth database was available from http://bioinfo.hrbmu.edu.cn/diseasemeth/. MethSurv database could provide the initial assessment of methylation-based cancer biomarkers [38]. MethSurv database was available from https://biit.cs.ut.ee/methsurv. In this study, we used DiseaseMeth database to analysis the DNA methylation level of SOX family. And we used MethSurv database to examine the effect of SOX family DNA methylation on patient survival time.

### 2.9. Statistical Analysis

Student' s *t*-test was used to analyze SOX family genes differentially expressed between HCC and normal tissues. The relationship between SOX family expression and clinicopathological features was analyzed through ANOVA. The correlations between SOX family genes were assessed using Pearson and Spearman correlation coefficients. In addition, survival analysis was performed using Kaplan-Meier analysis with the Log-rank test. We used *P* < 0.05 as statistical significance.

## 3. Results

### 3.1. Abnormal Expression Level of the SOX Family in HCC Patients

**Figure 1:**
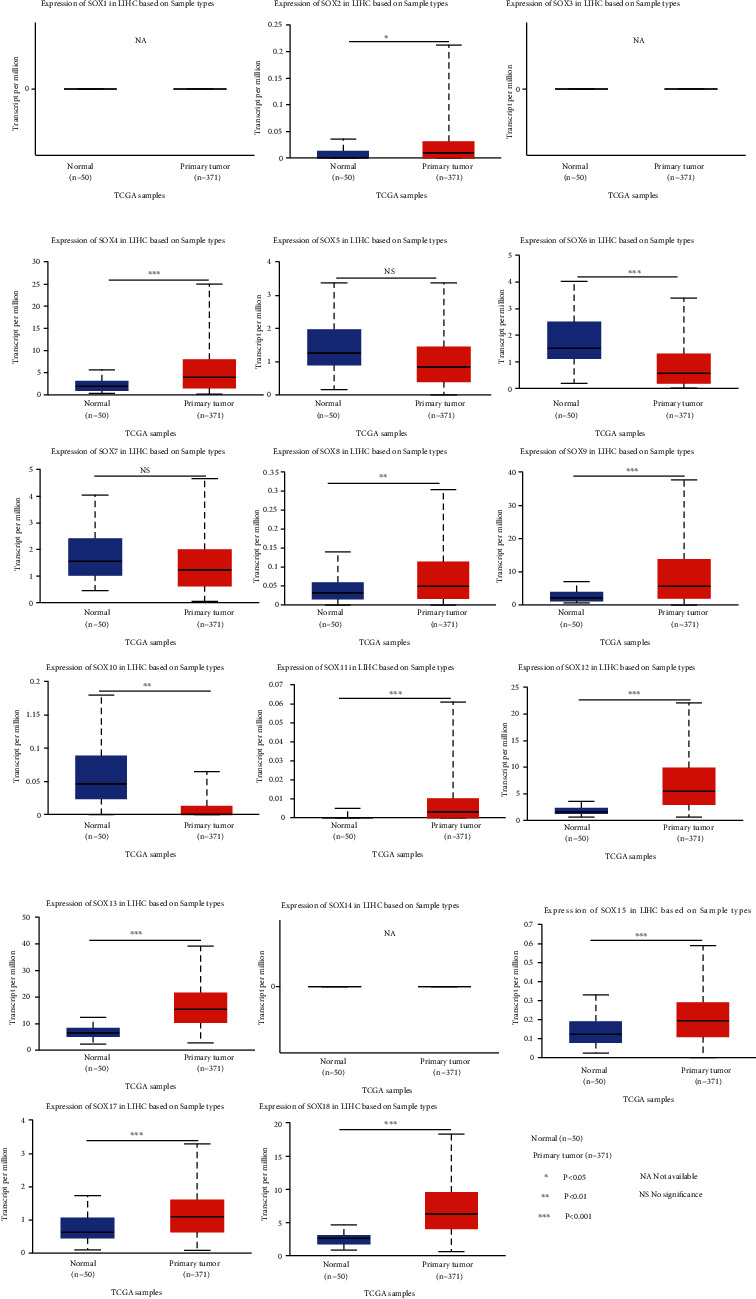
The mRNA expression profile of SOX family in HCC and normal tissues (UALCAN database). The mRNA expression level of SOX2, SOX4, SOX8, SOX9, SOX11, SOX12, SOX13, SOX15, SOX17, and SOX18 were higher in HCC than that in normal tissues. SOX6 and SOX10 had a lower expression in HCC than that in normal liver tissue.

UALCAN database was available to understand the mRNA expression level of SOX family between HCC and normal liver tissue. In this study, 371 HCC samples and 50 normal samples were used for analysis. And the mRNA expression level of SOX2, SOX4, SOX8, SOX9, SOX11, SOX12, SOX13, SOX15, SOX17, and SOX18 were significantly higher in HCC than that in normal tissues. In addition, SOX6 and SOX10 had a lower expression in HCC than that in normal liver tissue. Furthermore, the mRNA expression level of SOX5 and SOX7 had no significant statistical difference between HCC and normal liver tissue. To our surprise, the mRNA expression level of SOX1, SOX3, and SOX14 were not available in either HCC or normal liver tissue from the UALCAN database (Figure 1).

**Figure 2:**
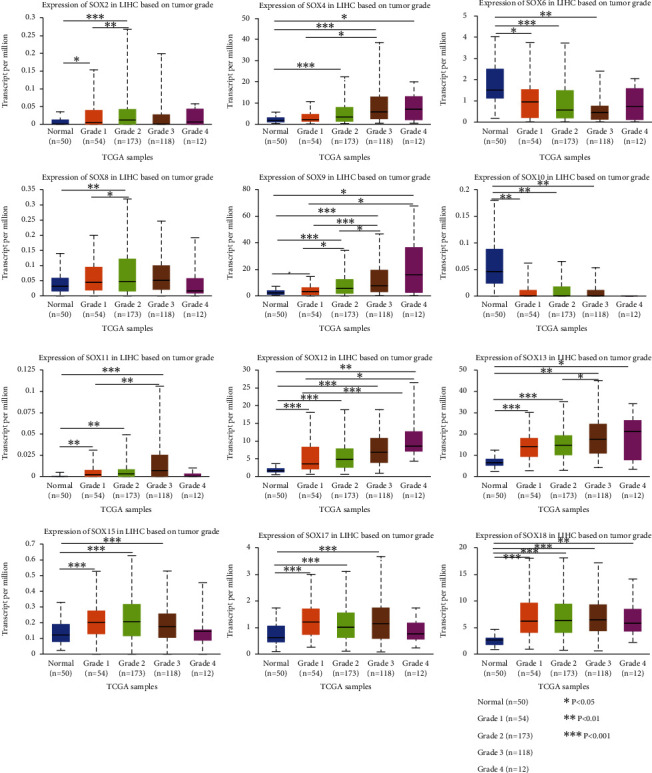
The relationship between SOX family mRNA expression and pathological stage of HCC patients (UALCAN database). The high mRNA expression level of SOX2, SOX4, SOX8, SOX9, SOX11, SOX12, SOX13, SOX15, SOX17, and SOX18 in HCC closely related to high-grade of tumor. SOX6 and SOX10 had a low expression in HCC, which also indicated high-grade of tumor.

Based on the differential expression of SOX family above, we then analyzed the consequence of these differentially expressed genes on the grade and metastasis of HCC patients in the UALCAN database. As is showed in Figure 2, there were 50 normal samples; in addition, 54 HCC samples were in grade 1, 173 HCC samples were in grade 2, 118 HCC samples were in grade 3, and 12 HCC samples were in grade 4. We found that the high mRNA expression level of SOX2, SOX4, SOX8, SOX9, SOX11, SOX12, SOX13, SOX15, SOX17, and SOX18 in HCC closely related to high grade of tumor. Moreover, SOX6 and SOX10 had a low expression in HCC, which also indicated high grade of tumor.

**Figure 3:**
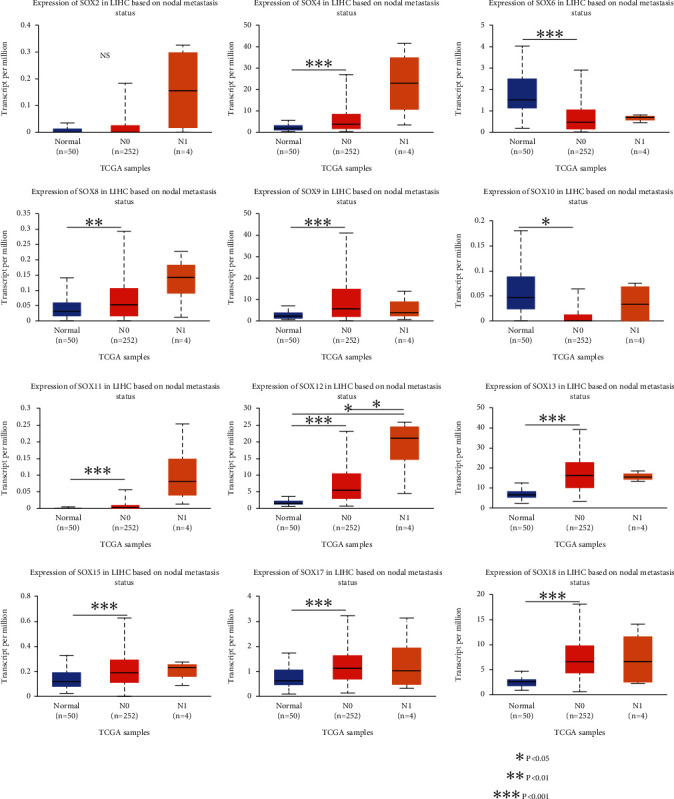
The relationship between SOX family mRNA expression and lymph node metastasis of HCC patients (UALCAN database). The high mRNA expression level of SOX4, SOX8, SOX9, SOX11, SOX12, SOX13, SOX15, SOX17, and SOX18 as well as the low mRNA expression level of SOX6 and SOX10 were positively associated with the N0 stage of HCC progression.

Subsequently, we analyzed the effect of differentially expressed genes of SOX family on HCC metastasis. All of these differentially expressed genes took part in the N0 progress of HCC, except SOX2. The high mRNA expression level of SOX4, SOX8, SOX9, SOX11, SOX12, SOX13, SOX15, SOX17, and SOX18 as well as the low mRNA expression level of SOX6 and SOX10 indicated HCC N0 progress. However, only the high mRNA expression level of SOX12 showed lymph node metastasis trends in HCC (Figure 3). The reason why there were few data to indicate the correlations between SOX family and the metastasis of multiple lymph node in HCC may be the inadequate sample size.

### 3.2. Prognostic Value of mRNA Expression of the SOX Family in HCC Patients

**Figure 4:**
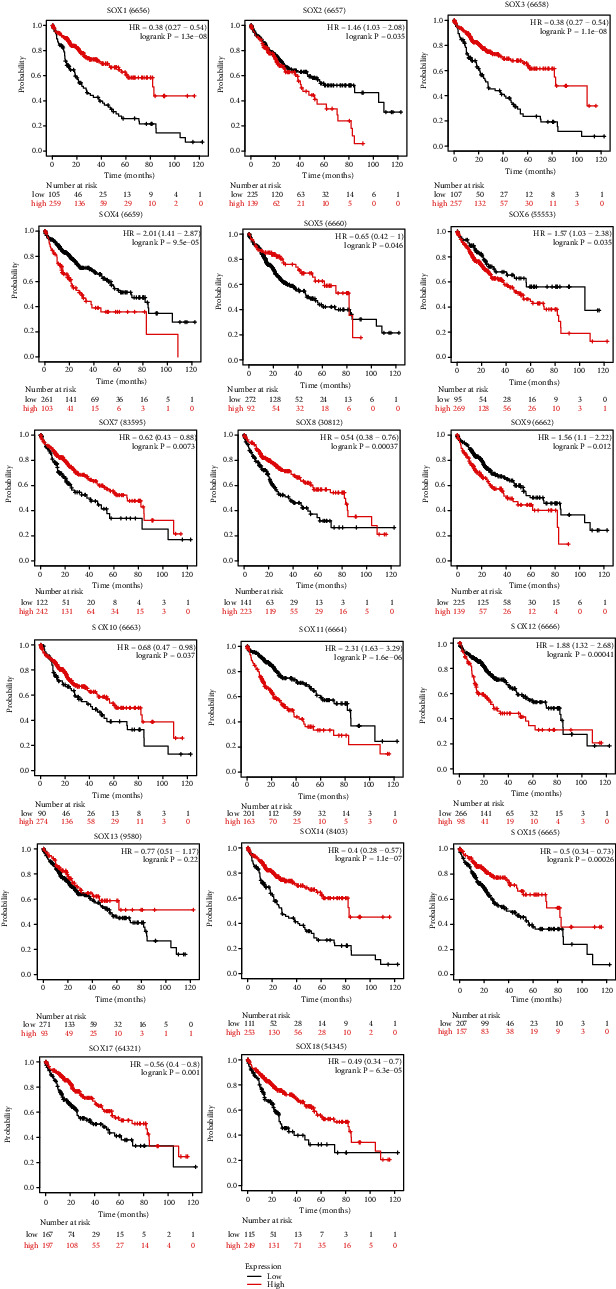
The overall survival curve of SOX family in HCC patients (Kaplan-Meier plotter database). All of the SOX family played a role in the OS of HCC patients except SOX13. SOX1, SOX3, SOX7, SOX10, SOX14, SOX15, SOX17, and SOX30 were negatively related with the OS of HCC. SOX2, SOX4, SOX6, SOX9, SOX11, and SOX12 showed positively relationship with OS time in HCC.

Next, we used Kaplan-Meier plotter to predict the prognostic values of mRNA expression level of SOX family in HCC patients. All of the SOX family played a role in the OS of HCC patients except SOX13. It was obvious that patients with higher mRNA transcription levels of SOX1, SOX3, SOX7, SOX10, SOX14, SOX15, and SOX17 displayed longer OS time in HCC. However, the higher mRNA transcription levels of SOX2, SOX4, SOX6, SOX9, SOX11, and SOX12 showed shorter OS time in HCC. Notably, the OS curve for the high mRNA expression level of SOX5, SOX8, SOX12, and SOX18 crossed with their OS curve at low mRNA expression level (Figure 4).

**Figure 5:**
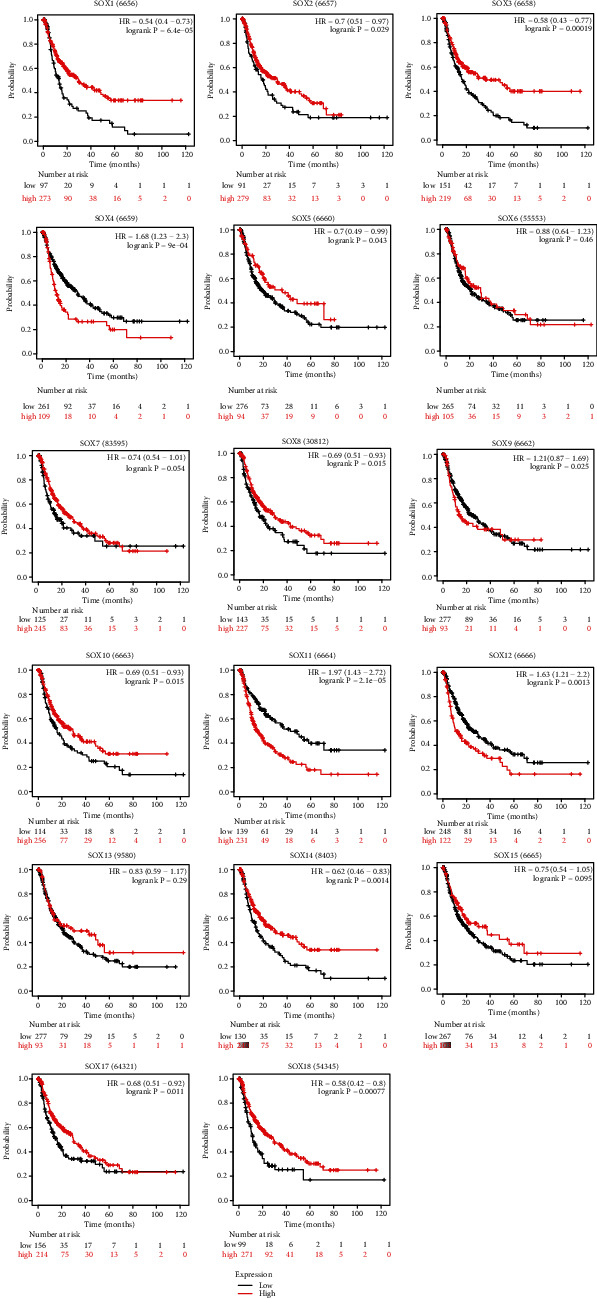
The progression free survival curve of SOX family in HCC patients (Kaplan-Meier plotter database). SOX1, SOX2, SOX3, SOX5, SOX8, SOX10, SOX14, and SOX18 were positively related with PFS time in HCC patients, while the high mRNA expression level of SOX4, SOX11, and SOX12 predicted poor PFS time in HCC.

In addition, the high mRNA expression level of SOX1, SOX2, SOX3, SOX5, SOX8, SOX10, SOX14, and SOX18 were positively related with PFS time in HCC patients, while the high mRNA expression level of SOX4, SOX11, and SOX12 predicted poor PFS time in HCC. In this database, the PFS curve for the high mRNA expression level of SOX17 crossed with its PFS curve at low mRNA expression level. And the mRNA expression level of SOX6, SOX7, SOX9, SOX13, and SOX15 showed no significant statistical difference in HCC patients' PFS (Figure 5).

### 3.3. Genetic Alteration of the SOX Family in HCC Patients

Based on the difference expression data results, we performed a comprehensive analysis of SOX family. We selected the genes that were significantly differentially expressed in HCC and normal tissues and were valuable for the grade and survival of HCC patients for the further investigation. So, we focused on SOX2, SOX4, SOX8, SOX10, SOX11, SOX12, SOX17, and SOX18. Next, to further analyze the functions of SOX family in HCC patients, we focused on the alteration profiles of the significantly differentially expressed genes above.

**Figure 6:**
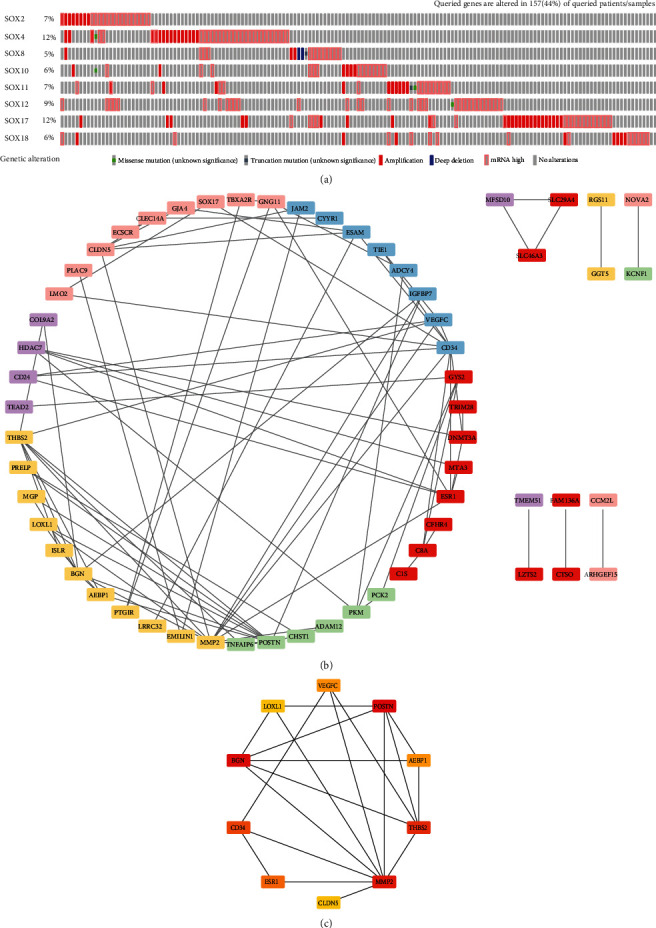
Genetic alteration and interaction analysis of the SOX family in HCC patients (cBioPortal database, String database, and Cytoscape software). We focused on SOX2, SOX4, SOX8, SOX10, SOX11, SOX12, SOX17, and SOX18. (a) All of these m6A “readers” had some genetic alteration, including “missense mutation,” “truncating mutation,” “amplification,” “deep deletion,” and “mRNA high.” Queried genes are altered in 157 (44%) of queried patients/samples. (b) PPI network used top 20 co-expression genes of each SOX family gene from cBioPortal database. This network was edited by STRING database and Cytoscape software. Different SOX family genes was depicted in different color (purple for SOX4, yellow for SOX8, green for SOX11, red for SOX12, blue for SOX17, and pink for SOX18). (c) The top 10 hub genes and their shortest paths of these co-expression genes. These data were analyzed by Cytoscape database.

We used cBioPortal database to check the genetic alterations of SOX family. As is displayed in Figure 6(a), all of these SOX family genes had some genetic alterations, which include “missense mutation,” “truncating mutation,” “amplification,” “deep deletion,” and “mRNA high.” Queried genes are altered in 157 (44%) of queried patients/samples. And among these alterations, “mRNA high” and “amplification” were the most common types. SOX4 and SOX17 had the highest mutation rate, with a mutation rate of up to 12%. In addition, the mutation rates of SOX12 (9%), SOX2 (7%), SOX11 (7%), SOX10 (6%), and SOX18 (6%) were no less than 5%.

### 3.4. Interaction Analysis of the SOX Family in HCC Patients

To discover the function of these differentially expressed SOX family, we further explored the top 20 co-expression molecules with high correlation (Spearman's correlation > 0.4) to each SOX gene through the cBioPortal database (Supplementary Table 1). However, among the co-expressed genes of SOX2 and SOX10, there was only one co-expression genes of SOX2 (SOX2-OT) and SOX10 (PLP1) which was eligible for filtering. We found that the Spearman's correlation of top 20 co-expression molecules of SOX8, SOX17, and SOX18 were more than 0.6. To understand the correlations among these co-expression molecules, we uploaded the eligible genes into the String database and then edited and modified them with Cytoscape software. Then, we build protein-protein interactions with these co-expression genes (Figure 6(b)) and picked out the top 10 hub genes as well as the shortest paths through cytoHubba plugin in Cytoscape software (Figure 6(c)). The top 10 hub genes were POSTN, BGN, MMP2, THBS2, CD34, ESR1, VEGFC, AEBP1, CLDN5, and LOXL1.

### 3.5. Functional Enrichment Analysis of the SOX Family in HCC Patients

**Figure 7:**
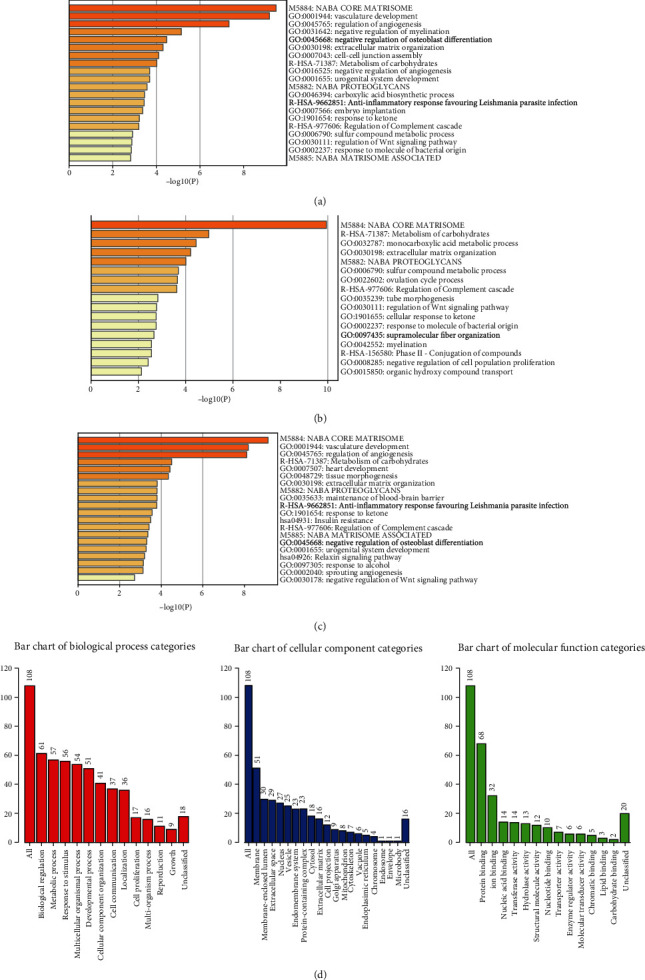
Functional enrichment analysis of the SOX family. (a) Bar plot of KEGG-enriched terms analyzed by Metascape. Co-expression genes of SOX family were enrichment in Naba core matrisome, vasculature development, regulation of angiogenesis, and negative regulation of myelination. (b) Functional enrichment analysis of SOX family co-expressed genes that were highly expressed in HCC and had a poor prognosis for patients and enriched them (they were SOX4, SOX11, and SOX12). These data were analyzed by Cytoscape database. (c) Functional enrichment analysis of SOX family co-expressed genes, which were highly expressed in HCC but had a good prognosis for patients. These data were analyzed by Cytoscape database. (d) GO enrichment analysis (molecular functions, biological processes, and cell components) of the co-expression genes of SOX family. These data were collected from WebGestalt database.

We re-analyzed all of these co-expression genes of SOX family in the PPI network through Metascape database. The results showed that co-expression genes were enrichment in Naba core matrisome, vasculature development, regulation of angiogenesis, and negative regulation of myelination (Figure 7(a)). In addition, we selected the SOX family co-expressed genes that were highly expressed in HCC and had a poor prognosis of patients and enriched them (including SOX4, SOX11, and SOX12) for further analysis. Naba core matrisome, metabolism of carbohydrates, monocarboxylic acid metabolic process, and extracellular matrix organization were the top pathways enriched (Figure 7(b)). Furthermore, SOX family co-expressed genes, which were highly expressed in HCC but had a good prognosis for patients, were also enriched for analysis. As is displayed, these genes were enriched in blood vessel development, Naba core matrisome, vasculature development, regulation of angiogenesis, and metabolism of carbohydrates (Figure 7(c)). To our surprise, the top 3 pathways enriched to these genes overlapped with 100 percent of the pathways enriched to all SOX family genes.

In this study, the WebGestalt database was used to analyze the biological functions of the SOX family. From the GO analysis, it was clearly that the most highly enriched biological process category was biological regulation, followed by metabolic process, response to stimulus, multicellular organismal process, development process, etc. In the cellular component categories, membrane, membrane-enclosed extracellular space, nucleus, vesicle, endomembrane system, protein-containing complex, and cytosol were highly enriched. As for the molecular function category, the co-expression genes of SOX family were mainly enriched in protein binding, ion binding, nucleic acid binding, and transferase activity (Figure 7(d)).

### 3.6. Immune Cell Infiltration of SOX Family in HCC Patients

**Figure 8:**
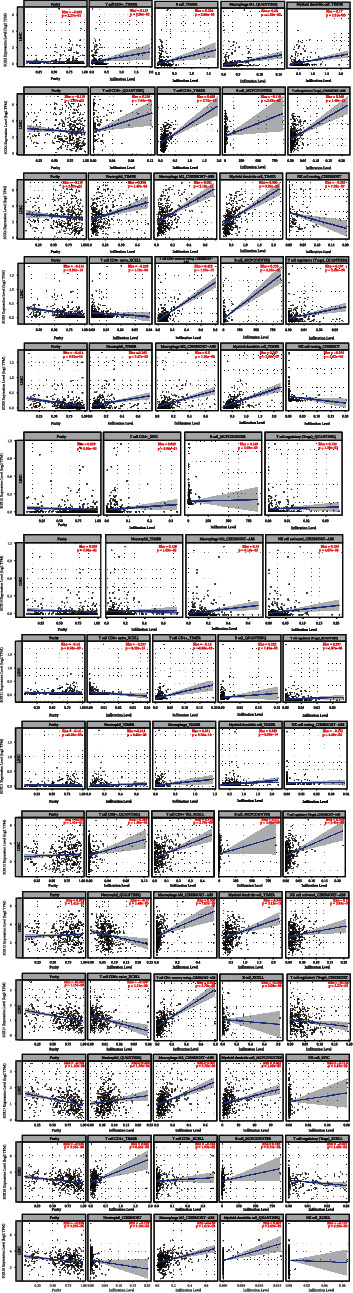
Immune cell infiltration of the SOX family. We searched the correlation among 8 types of immune cells (including CD8+ T cell, CD4+ T cell, B cell, Tregs, neutrophil, macrophage, myeloid DC, and NK cell) and SOX family through the TIMER 2.0 database. SOX4, SOX8, SO11, SOX12, SOX17, and SOX18 were correlation with 8 types of immune cells above.

We searched the correlation among 8 types of immune cells (including CD8+ T cell, CD4+ T cell, B cell, Tregs, neutrophil, macrophage, myeloid DC, and NK cell) and SOX family through the TIMER 2.0 database. In this study, all of the results had a purity adjustment to avoiding confounding factor in immune cell infiltration analysis. As is showed in Figure 8, SOX4, SOX8, SO11, SOX12, SOX17, and SOX18 were correlation with 8 types of immune cells above. SOX2 had a positive correlation with CD4+ T cell, B cell, macrophage, and myeloid DC, while the correlation between this gene and CD8+ T cell, Tregs, neutrophil, as well as NK cell had no significant statistical difference. In addition, SOX10 had a positive correlation with CD4+ T cell, B cell, Tregs, neutrophil, macrophage, as well as NK cell, while the correlation between this gene and CD8+ T cell and myeloid DC had no significant statistical difference.

From this database, we discovered that most types of immune cells had a positive correlation with SOX family. Notably, CD4+ T cell and macrophage were positively related with all these SOX family, while NK cells were negatively related with most SOX family genes including SOX4, SOX8, SOX11, SOX17, and SOX18. Moreover, Tregs were negatively related with SOX11, SOX17, and SOX18. CD8+ T cell was negatively related with SOX8, SOX17, and SOX18. Neutrophil was negatively related with SOX17 and SOX18. However, the correlation between immune cells and SOX10 as well as SOX11 seems not remarkable.

### 3.7. Methylation Levels of SOX Family in HCC Patients

**Figure 9:**
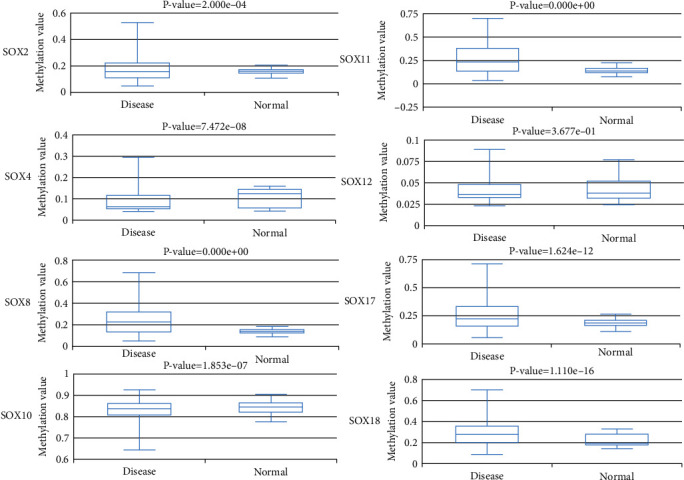
Methylation levels of SOX family in HCC Patients. The DNA methylation levels in promoter area of SOX2, SOX4, SOX10, and SOX30 were lower in HCC than normal tissues. While SOX8, SOX11, SOX17, and SOX18 had higher DNA methylation levels than normal tissues. These data were collected from DiseaseMeth database.

We analyzed the methylation levels of SOX family by searching the DiseaseMeth database. We found that the DNA methylation levels in promoter area of SOX2, SOX4, and SOX10 were lower in HCC than normal tissues, while SOX8, SOX11, SOX17, and SOX18 had higher DNA methylation levels than normal tissues. However, the DNA methylation levels in promoter area of SOX12 seem no significant statistical difference between HCC and normal tissue (Figure 9).

**Figure 10:**
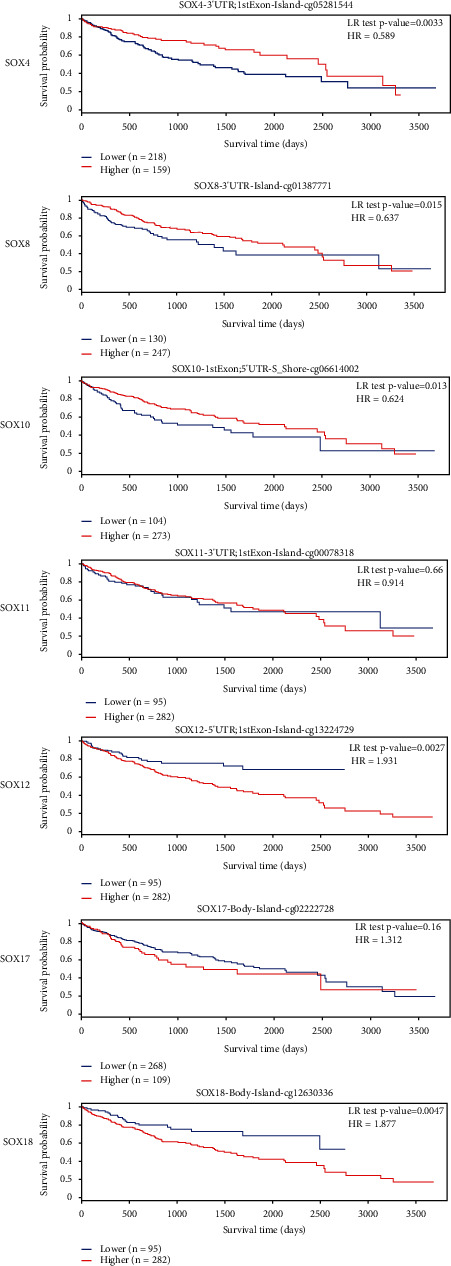
The survival information about the DNA methylation level of SOX family in HCC Patients. The higher DNA methylation level of SOX12 and SOX18 demonstrated better survival rates in patients with HCC. Hypermethylation of the SOX4, SOX8, and SOX10 gene overlapped with the survival curve of hypomethylation HCC patients, but in the early stage of HCC development, hypermethylation of SOX4, SOX8, and SOX10 gene could indicate a better prognosis.

In addition, we put these SOX family into MethSurv database for further analysis. However, the survival information about the DNA methylation level of the SOX2 was not available in the database. Moreover, the DNA methylation level of SOX11 and SOX17 seems no significant statistical difference between HCC and normal tissue. The higher DNA methylation level of SOX12 and SOX18 demonstrated lower survival rates in patients with HCC. Hypermethylation of the SOX4, SOX8, and SOX10 gene overlapped with the survival curve of hypomethylation HCC patients, but in the early stage of HCC development, hypermethylation of the SOX4, SOX8, and SOX10 genes could indicate a better prognosis (Figure 10).

### 3.8. Validation of the Expression of SOX Gene with the Highest Alteration Rate in the Tissue and Cell Lines of HCC and Normal Liver

**Figure 11:**
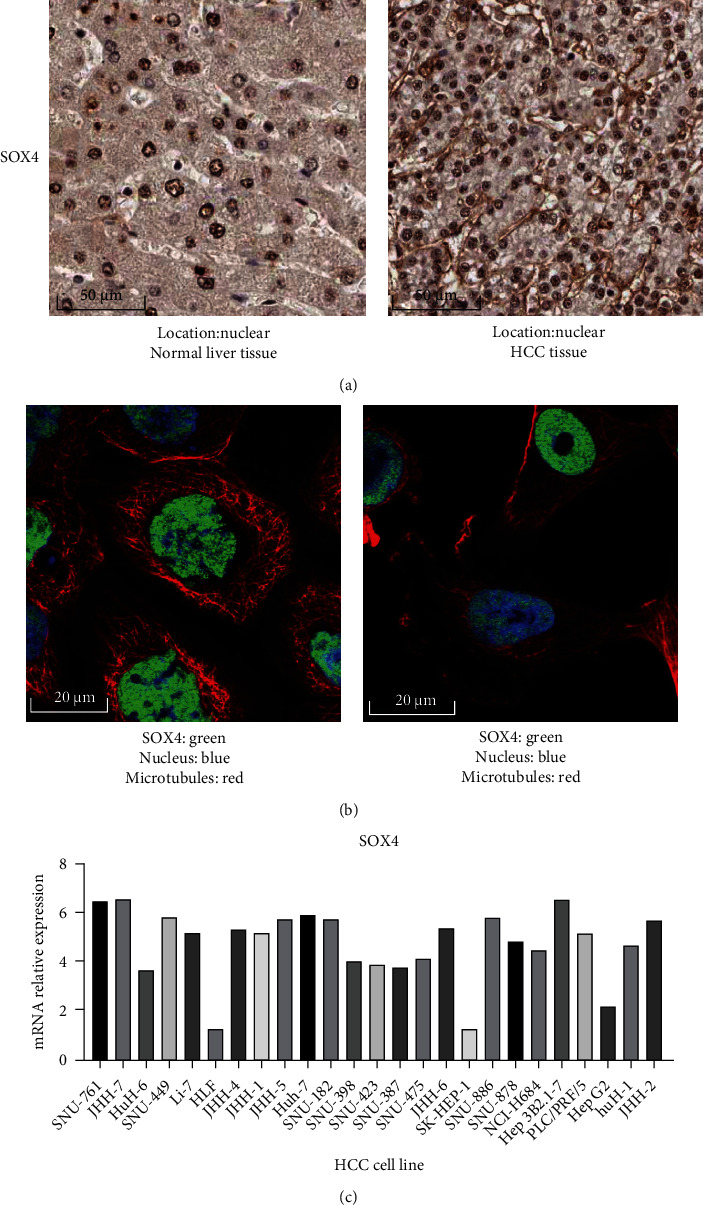
Validation of the expression of SOX gene with the highest alteration rate in the tissue and cell lines of HCC and normal liver. (a) The protein expression level of SOX4 in HCC tissue and in normal liver tissue. This data was derived from HPA database. (b) The immunofluorescence results of SOX4. SOX4 was localized in the nucleus. (SOX4 was stained green, the nucleus was stained blue, and the microtubules were stained red.) This data was derived from HPA database. (c) The mRNA expression level of SOX4 in HCC cell lines. This data was derived from CCLE database.

To verify the reliability of the conclusions, SOX4 was selected for further analysis because it had the highest rate of genetic alteration (12%). First, we would like to explore the protein expression information of SOX4 in the tissue level. So, the HPA database was used. As is showed in Figure 11(a), SOX4 was expressed at nuclear both in HCC and normal liver tissue, and the protein expression level of SOX4 in HCC was higher than that in normal liver tissue. In addition, we further demonstrated that SOX4 was localized in the nucleus by immunofluorescence results (Figure 11(b)). As is shown in the picture, SOX4 was stained green, the nucleus was stained blue, and the microtubules were stained red. The immunofluorescence information above were based on A-431 (Figure 11(b) left) and U-251 (Figure 11(b) right) cell lines. We then further explored the gene expression level of SOX4 at the cellular level. CCLE database was used to check the mRNA expression level of SOX4 in HCC cell lines (Figure 11(c)). And it seems that the mRNA expression level of SOX4 was highly expressed in most of HCC cell lines.

## 4. Discussion

SOX family are transcription factors that could regulate different molecular pathways and their expression. SOX family members have previously been considered to be involved in the regulation of human cancer. For example, in glioma, numerous SOX family were reported to play a significant role in the initiation of glioma cells differentiation, and almost all SOX family were expressed in GBM, and their mRNA expression levels were associated with glioma patient's prognosis [39]. Moreover, SOX transcription factors such as SOX9 could also play a double-edged sword role in cervical cancer. Study showed it could be both tumor-suppressor and tumor-promoting factor in cervical cancer [40]. In addition, PITX1 inhibited the development and progression of melanoma through targeting of the SOX signaling [15]. However, the integrated comprehensive information, detailed functions, and mechanisms of these SOX family in HCC have not been fully explored and explained so far.

In this research, the SOX family were comprehensively analyzed based on their mRNA expression levels as well as the clinical prognostic value in HCC. Then, we focused on SOX2, SOX4, SOX8, SOX10, SOX11, SOX12, SOX17, and SOX18, because these genes were significantly differentially expressed in HCC and normal tissues and they were also valuable for the grade and survival of HCC patients. Reviewing the previous studies, we found that these genes were reported to play a significant role in HCC, which verified our bioinformatics analysis results. For instance, silenced SOX2 gene expression could reduce the growth rate of HCC xenografts and enhance the therapeutic response in HCC [41, 42]. SOX4 was reported to modulate the endothelial cell migration and angiogenesis in HCC, and it could act as a biomarker in hepatitis B virus-associated HCC [43–46]. SOX8 was significantly upregulated in HCC and its upregulation promoted cancer cell proliferation in HCC [47]. SOX11 was reported to have a potential to regulate the apoptosis and cell cycle in HCC through Wnt/*β*-catenin signaling pathway [48]. SOX12 acted as a cancer stem-like cell marker and could promote malignant phenotypes of HCC, such as metastasis [49–51]. SOX17 inhibited the Wnt/*β*-catenin signaling pathway and thus inhibited the growth of HCC cells [52]. SOX18 knockdown significantly reduced FGF19-enhanced HCC invasion and metastasis. SOX18 increased the HCC cell viability, migration, invasiveness, and decreased apoptosis in HCC through FGF19-SOX18-FGFR4 positive feedback loop and AMPK/mTOR signaling pathway [25, 53, 54].

It is well known that mutations usually lead to abnormal cell function or death, and in advanced organisms can even lead to cancer. To further analyze the functions of SOX family in HCC patients, we focused on the alteration profiles of these genes above. And to our surprise, all of these SOX family genes had some genetic alterations. That is to say, the gene alteration of SOX2, SOX4, SOX8, SOX10, SOX11, SOX12, SOX17, and SOX18 may provide some information for the progression of HCC.

Next, we constructed a protein-protein interaction network for the co-expressed genes of the SOX family and identified the hub genes in this network to better understand the functions and patterns of the SOX family. Results revealed that among all SOX family proteins, SOX8, SOX17, and SOX18 showed correlation coefficients greater than 0.6 with their co-expressed molecules, which may suggest that their roles in HCC may be closely related to the co-expressed molecules. The co-expression molecules of SOX2 and SOX10 were really small, so the roles of these three SOX family molecules in HCC may need to be explored separately. In addition, the top 10 hub genes POSTN, BGN, MMP2, THBS2, CD34, ESR1, VEGFC, AEBP1, CLDN5, and LOXL1 seem to be related with cancer stemness (such as cancer-associated fibroblasts [55], EMT process [56], tumor immune infiltration [57], and metastasis [58]). In conclusion, the function of SOX family on HCC may link with these cancer stemness-related proteins, and these proteins may have a potential to be biomarkers or therapeutic targets.

We conducted enrichment analysis of SOX family co-expressed genes, and the results demonstrated that enrichment analysis of SOX family co-expressed genes that are highly expressed in HCC but have a good prognosis for patients overlaps with 80% enrichment to all SOX family genes in the pathway. According to the enrichment analysis, regulation of angiogenesis associated pathway was the most enriched.

A recent study showed some new evidence that SOX family can regulate the tumor immune microenvironment [19]. So, we analyzed the correlation among 8 types of immune cells (including CD8+ T cell, CD4+ T cell, B cell, Tregs, neutrophil, macrophage, myeloid DC, and NK cell) and SOX family through the TIMER 2.0 database. And the results demonstrated that SOX4, SOX8, SO11, SOX12, SOX17, and SOX18were correlation with 8 types of immune cells above. And the therapeutic efficacy of immunotherapy and tumor progression could modulate by the composition and abundance of immune cells in the tumor microenvironment. That may indicate that the function of these SOX families in HCC is likely to be closely related to immune cell infiltration. In addition, most types of immune cells (especially CD4+ T cell and macrophage) had a positive correlation with SOX family. CD4+ T cell is an important immune cell in human immune system; it is mainly expressed in helper T (Th) cells and played an indispensable role in tumor immunity [59]. Research showed that during the Th2 cell differentiation process, SOX12 mRNA was significantly increased [60]. Macrophages are specialized cells of the natural immune system with long survival time and phagocytosis. Macrophages also appear to have a critical role in the tumor microenvironment, particularly in stromal remodeling, angiogenesis, metastasis, and tumor progression [61]. Tumor-associated macrophages upregulated the expression of SOX2 and promoted CSC-like phenotypes in breast cancer cells [62]. However, there are very few literature reports on the interaction between SOX family and immune infiltration in HCC, and it may be a novel insight in the near future.

Moreover, the DNA sequences methylation values of certain SOX family may be a promising factor for HCC diagnostics. DNA methylation is a chemical modification of DNA that can alter genetic expression without altering the DNA sequence. The DNA methylation levels in promoter area of the analyzed SOX family were differed between HCC and normal tissues except for SOX12. However, when we proceeded to the mentioned DNA methylation levels of the SOX family on the prognosis of patients with HCC, hypomethylation expression of SOX12 had a better prognosis for patients. This may be as a consequence of differences in calculation methods between databases or owing to the differences on methylation modification sites. In addition, we found that DNA methylation levels of SOX4, SOX8, SOX10, and SOX18 also had an impact on the survival of patients with HCC. In this study, we identified some DNA methylation modification sites (the 3′UTR region of SOX4 and SOX8, 5′UTR region of SOX10 and SOX12, and body island region of SOX18) in the SOX family that had an impact on the survival of patients with HCC. However, more detailed information about the relationship between DNA methylation modification sites and the survival of HCC needs to be elucidated by further research.

In general, DNA methylation usually acts as an inhibitory factor of gene transcription when the methylation is located in the promoter region. Hypermethylation of gene leads to low expression of mRNA. Therefore, we integrated the DNA methylation level with the mRNA expression level of SOX family in HCC. However, in SOX family genes, the correlation between methylation levels and mRNA expression levels seems to not completely follow this pattern exactly, with the exception of SOX2 and SOX10. In fact, different studies have identified other new patterns of DNA methylation and revealed the role of complex and diverse epigenetic landscapes in genomes. Meromit Singer and his colleges declared that intragenic methylation was found to be positively correlated with gene expression, and the exons were more highly methylated than their neighboring introns. However, they also identified a subset of hypomethylated exons that displayed lower methylation levels than their surrounding introns. And they observed a negative correlation between exon methylation and the density of most histone modifications [63]. In addition, previous researches have steadily integrated a more widespread understanding that methylation patterns of intragenic or gene body methylation may function in transcriptional regulation and efficiency. Intragenic methylation could suppress repetitive element transcription, and the methylated intragenic regions were related to higher levels of gene transcription [64].For example, in melanoma samples with high MMP-9 transcript levels, the DNA of the intragenic CpG-2 region of the MMP9 gene was highly methylated, and high mRNA and protein levels of MMP-9 in this region were verified in vitro [65]. Although these findings contribute to the understanding of the relationship between methylation levels in the SOX family and the gene expression levels as well as survival of patients with HCC, the specific mechanisms need further investigation.

In our further analysis and validation of SOX4 expression, we found that SOX4 showed high expression in most of HCC cell lines according to the CCLE database, which was consistent with the results of SOX4 expression levels in the UALCAN database.

## 5. Conclusions and limitations

In conclusion, our integrative analysis of SOX family uncovered the associations of SOX family expression with survival outcomes, mutation situation, functional enrichment analysis, immune cells infiltration, and methylation levels of the SOX genes in HCC, which could facilitate the explanation of the functions of SOX family in carcinogenesis, immunotherapeutic response, and epigenetics level from various perspectives. However, all of the data in this article are derived from public databases, and more experiments were required to verify the biological functions of important SOX molecules.

## Figures and Tables

**Table 1:** Clinical information of HCC patients in The Cancer Genome Atlas (TCGA) database.

Clinical features	Variables	Total (*n* = 371)	Percentages (%)
Age	≤60	167	45
	>60	191	51
	Unknown	13	4
Gender	Female	117	32
	Male	245	66
	Unknown	9	2
Tumor grade	G1	54	14
	G2	173	47
	G3	118	32
	G4	12	3
	Unknown	14	4
Individual cancer stage	Stage 1	168	45
	Stage 2	84	23
	Stage 3	82	22
	Stage 4	6	16
	Unknown	31	84
Nodal metastasis status	N0	252	68
	N1	4	1
	Unknown	115	31

## Data Availability

All data generated or analyzed during this study are included in this article.

## Abbreviations

BP: Biological process
CC: Cellular component
CCLE: Cancer cell line encyclopedia
DC: Dendritic cell
GO: Gene ontology
GSEA: Gene set enrichment analysis
HPA: The human protein atlas
KEGG: Kyoto Encyclopedia of Genes and Genomes
HCC: Hepatocellular carcinoma
HPA: Human protein atlas
Th: Helper T
MF: Molecular function
NK: Nature killer
NTA: Network topology-based analysis
ORA: Over-representation analysis
OS: Overall survival
PFS: Progression-free survival
PPI: Protein-protein interactions
SOX: Sex-determining region Y (Sry)-box-containing
Tregs: T cell regulatory.

## Supplementary Materials

Supplementary Materials
Supplementary Table 1: The top 20 co-expression molecules with high correlation (Spearman's correlation >0.4) to each SOX gene through the cBioPortal database.
