# Supplementary material for: The Dysregulation of SOX Family Correlates with DNA Methylation and Immune Microenvironment Characteristics to Predict Prognosis in Hepatocellular Carcinoma
Source: Dis Markers. 2022 Apr 13;2022:2676114. doi: 10.1155/2022/2676114 (PMC9020970; doi:10.1155/2022/2676114)
Supplement: Supplementary Materials — Supplementary Table 1: The top 20 co-expression molecules with high correlation (Spearman's correlation >0.4) to each SOX gene through the cBioPortal database. [file 2676114.f1.docx]

| **Supplementary Table1. Co-expression genes of SOX family genes (Spearman's Correlation>0.4)** | | | |
| --- | --- | --- | --- |
| SOX family genes | Correlated Gene | Spearman's Correlation | p-Value |
| SOX2 | SOX2-OT | 0.475524564 | 1.04E-21 |
| SOX4 | MFSD10 | 0.660563739 | 1.72E-46 |
| SOX4 | TMEM132A | 0.644475137 | 1.23E-43 |
| SOX4 | PKM | 0.626590483 | 1.16E-40 |
| SOX4 | TNFRSF21 | 0.62349298 | 3.65E-40 |
| SOX4 | DBN1 | 0.622728828 | 4.83E-40 |
| SOX4 | DCXR | -0.622151148 | 5.96E-40 |
| SOX4 | PCK2 | -0.615730574 | 6.04E-39 |
| SOX4 | MTMR2 | 0.614538692 | 9.23E-39 |
| SOX4 | RNF24 | 0.613727215 | 1.23E-38 |
| SOX4 | HAGH | -0.610651317 | 3.64E-38 |
| SOX4 | COL9A2 | 0.610581307 | 3.73E-38 |
| SOX4 | CD24 | 0.60792753 | 9.40E-38 |
| SOX4 | WNK2 | 0.60786664 | 9.60E-38 |
| SOX4 | HDAC7 | 0.606184719 | 1.72E-37 |
| SOX4 | TEAD2 | 0.603598536 | 4.17E-37 |
| SOX4 | CASC15 | 0.600857645 | 1.06E-36 |
| SOX4 | MARCKSL1 | 0.59987397 | 1.48E-36 |
| SOX4 | TMEM51 | 0.599508227 | 1.67E-36 |
| SOX4 | AASS | -0.598417683 | 2.41E-36 |
| SOX4 | ACSM2B | -0.596480168 | 4.60E-36 |
| SOX8 | AEBP1 | 0.746493009 | 2.59E-65 |
| SOX8 | LOXL1 | 0.740016106 | 1.23E-63 |
| SOX8 | BGN | 0.72794996 | 1.21E-60 |
| SOX8 | GGT5 | 0.722793094 | 2.05E-59 |
| SOX8 | ISLR | 0.721404902 | 4.35E-59 |
| SOX8 | PTGIR | 0.718590545 | 1.97E-58 |
| SOX8 | SSC5D | 0.717077741 | 4.40E-58 |
| SOX8 | PDZD4 | 0.714038667 | 2.18E-57 |
| SOX8 | PRELP | 0.712459802 | 4.96E-57 |
| SOX8 | THBS2 | 0.711756077 | 7.15E-57 |
| SOX8 | RGS11 | 0.711433242 | 8.45E-57 |
| SOX8 | PODN | 0.710979683 | 1.07E-56 |
| SOX8 | TMEM132E | 0.705051705 | 2.19E-55 |
| SOX8 | LRRC32 | 0.704054961 | 3.62E-55 |
| SOX8 | DACT3 | 0.703083059 | 5.88E-55 |
| SOX8 | EMILIN1 | 0.701099099 | 1.58E-54 |
| SOX8 | FNDC1 | 0.700857364 | 1.78E-54 |
| SOX8 | MGP | 0.699831309 | 2.95E-54 |
| SOX8 | MMP2 | 0.697789357 | 8.02E-54 |
| SOX8 | AGAP2-AS1 | 0.69674213 | 1.34E-53 |
| SOX10 | PLP1 | 0.433014786 | 6.94E-18 |
| SOX11 | PLXDC1 | 0.601834795 | 7.60E-37 |
| SOX11 | FOXL1 | 0.583622476 | 3.03E-34 |
| SOX11 | N4BP3 | 0.580349973 | 8.56E-34 |
| SOX11 | BMP8A | 0.555091979 | 1.75E-30 |
| SOX11 | DCXR | -0.54581795 | 2.45E-29 |
| SOX11 | ADAM12 | 0.545583092 | 2.61E-29 |
| SOX11 | HTRA3 | 0.545109321 | 2.98E-29 |
| SOX11 | RNF24 | 0.544594529 | 3.44E-29 |
| SOX11 | CHST1 | 0.539974555 | 1.24E-28 |
| SOX11 | PKM | 0.534758728 | 5.12E-28 |
| SOX11 | TNFAIP6 | 0.522566028 | 1.29E-26 |
| SOX11 | UNC5B | 0.521927868 | 1.52E-26 |
| SOX11 | APCDD1L | 0.518463991 | 3.70E-26 |
| SOX11 | POSTN | 0.517185822 | 5.13E-26 |
| SOX11 | PEBP1 | -0.51684416 | 5.60E-26 |
| SOX11 | SLC27A5 | -0.516026967 | 6.89E-26 |
| SOX11 | FOXF2 | 0.515240743 | 8.41E-26 |
| SOX11 | KCNF1 | 0.51405115 | 1.14E-25 |
| SOX11 | PCK2 | -0.51182875 | 1.98E-25 |
| SOX11 | FHDC1 | 0.511822927 | 1.99E-25 |
| SOX12 | DNMT3A | 0.59608176 | 5.26E-36 |
| SOX12 | LZTS2 | 0.577304352 | 2.22E-33 |
| SOX12 | C1S | -0.567415901 | 4.60E-32 |
| SOX12 | FAM136A | 0.565553747 | 8.05E-32 |
| SOX12 | ESR1 | -0.563064309 | 1.69E-31 |
| SOX12 | PAFAH1B3 | 0.562107218 | 2.25E-31 |
| SOX12 | GLYATL1 | -0.550766888 | 6.04E-30 |
| SOX12 | DLK2 | 0.545510245 | 2.67E-29 |
| SOX12 | SLC46A3 | -0.538012382 | 2.12E-28 |
| SOX12 | GYS2 | -0.536632484 | 3.08E-28 |
| SOX12 | COPS7B | 0.533230966 | 7.72E-28 |
| SOX12 | CTSO | -0.531002297 | 1.40E-27 |
| SOX12 | SLC27A2 | -0.529084072 | 2.33E-27 |
| SOX12 | CFHR4 | -0.523677346 | 9.65E-27 |
| SOX12 | KLF9 | -0.523345602 | 1.05E-26 |
| SOX12 | TRIM28 | 0.522907584 | 1.18E-26 |
| SOX12 | MTA3 | 0.522426871 | 1.34E-26 |
| SOX12 | SLC29A4 | 0.519519698 | 2.83E-26 |
| SOX12 | C8A | -0.519336055 | 2.96E-26 |
| SOX12 | C3P1 | -0.513379107 | 1.35E-25 |
| SOX17 | CLEC14A | 0.816597865 | 1.86E-87 |
| SOX17 | CCM2L | 0.802243587 | 3.29E-82 |
| SOX17 | ARHGEF15 | 0.797900447 | 1.05E-80 |
| SOX17 | TIE1 | 0.794380358 | 1.63E-79 |
| SOX17 | GJA4 | 0.793650928 | 2.87E-79 |
| SOX17 | ADCY4 | 0.786828345 | 4.94E-77 |
| SOX17 | NOVA2 | 0.781736485 | 2.04E-75 |
| SOX17 | JAM2 | 0.778702511 | 1.79E-74 |
| SOX17 | MYCT1 | 0.776824667 | 6.72E-74 |
| SOX17 | CYYR1 | 0.770689846 | 4.68E-72 |
| SOX17 | ESAM | 0.770177496 | 6.63E-72 |
| SOX17 | RAMP2 | 0.769439065 | 1.09E-71 |
| SOX17 | HSPA12B | 0.768628616 | 1.89E-71 |
| SOX17 | ECSCR | 0.762134739 | 1.40E-69 |
| SOX17 | TBXA2R | 0.761006901 | 2.92E-69 |
| SOX17 | USHBP1 | 0.760759985 | 3.42E-69 |
| SOX17 | FAM110D | 0.754197789 | 2.25E-67 |
| SOX17 | VEGFC | 0.751306257 | 1.36E-66 |
| SOX17 | CD34 | 0.74493759 | 6.61E-65 |
| SOX17 | IGFBP7 | 0.744260115 | 9.92E-65 |
| SOX18 | NOVA2 | 0.719969804 | 9.42E-59 |
| SOX18 | GNG11 | 0.707765235 | 5.55E-56 |
| SOX18 | INKA1 | 0.692013357 | 1.30E-52 |
| SOX18 | FAM110D | 0.68982537 | 3.68E-52 |
| SOX18 | ECSCR | 0.687415026 | 1.14E-51 |
| SOX18 | LMO2 | 0.674536069 | 4.07E-49 |
| SOX18 | USHBP1 | 0.670096477 | 2.88E-48 |
| SOX18 | RAMP2 | 0.669838502 | 3.22E-48 |
| SOX18 | CCM2L | 0.668983043 | 4.68E-48 |
| SOX18 | FAM167B | 0.656992766 | 7.64E-46 |
| SOX18 | ARHGEF15 | 0.656572967 | 9.10E-46 |
| SOX18 | ID3 | 0.654615648 | 2.04E-45 |
| SOX18 | EXOC3L1 | 0.651321384 | 7.86E-45 |
| SOX18 | GIPC3 | 0.649034843 | 1.98E-44 |
| SOX18 | PLAC9 | 0.647045888 | 4.41E-44 |
| SOX18 | TBXA2R | 0.646894652 | 4.68E-44 |
| SOX18 | SOX17 | 0.646405451 | 5.69E-44 |
| SOX18 | GJA4 | 0.645411359 | 8.45E-44 |
| SOX18 | CLEC14A | 0.645160328 | 9.34E-44 |
| SOX18 | CLDN5 | 0.64404072 | 1.46E-43 |
